# Identifying Cancer Type-Specific Transcriptional Programs through Network Analysis

**DOI:** 10.3390/cancers15164167

**Published:** 2023-08-18

**Authors:** Jiji T. Kurup, Seongho Kim, Benjamin L. Kidder

**Affiliations:** 1 Department of Oncology, Wayne State University School of Medicine, Detroit, MI 48201, USA; jijikurup@gmail.com (J.T.K.); kimse@karmanos.org (S.K.); 2 Karmanos Cancer Institute, Wayne State University School of Medicine, Detroit, MI 48201, USA

**Keywords:** cancer, gene regulatory networks, network biology

## Abstract

**Simple Summary:**

This study focused on identifying cancer type-specific genes, which are crucial for improving the detection, diagnosis, and treatment of various types of cancer. We used a network biology framework to explore the expression of transcription factors in different types of cancer. By comparing gene networks in normal cells with those in cancer cells, we were able to identify cancer type-specific genes. This offers a resource for understanding transcriptional networks across various cancer types.

**Abstract:**

Identifying cancer type-specific genes that define cell states is important to develop effective therapies for patients and methods for detection, early diagnosis, and prevention. While molecular mechanisms that drive malignancy have been identified for various cancers, the identification of cell-type defining transcription factors (TFs) that distinguish normal cells from cancer cells has not been fully elucidated. Here, we utilized a network biology framework, which assesses the fidelity of cell fate conversions, to identify cancer type-specific gene regulatory networks (GRN) for 17 types of cancer. Through an integrative analysis of a compendium of expression data, we elucidated core TFs and GRNs for multiple cancer types. Moreover, by comparing normal tissues and cells to cancer type-specific GRNs, we found that the expression of key network-influencing TFs can be utilized as a survival prognostic indicator for a diverse cohort of cancer patients. These findings offer a valuable resource for exploring cancer type-specific networks across a broad range of cancer types.

## 1. Introduction

Cellular fate transitions are essential to ensure faithful differentiation and development, where lineage specification is tightly controlled by transcription factor (TF) activity and chromatin organization. Sequence-specific TFs and chromatin modifying enzymes provide constraints to dictate diverse gene expression programs during development. In this highly choreographed process, TFs bind sets of genes that define cell type-specific gene regulatory networks (GRN), which are conditionally expressed following chromatin remodeling. The processes that are critical to maintaining normal cellular function can be disrupted or hijacked during tumorigenesis or neoplastic states by aberrantly controlling transcription. Overlapping gene expression patterns between normal and cancer cells and activation of developmental *cis*-regulatory elements in cancer cells suggest a shared epigenetic state, where epigenetic reprogramming establishes permissive or restrictive chromatin environments that facilitate tumorigenesis.

Directed in vitro differentiation of stem cells has been a focus of biologists to derive cell types for the purpose of studying development, regenerative medicine, and drug screening. An assortment of lineages have been reprogrammed to an induced pluripotent (iPS) stem cell state [1,2,3,4,5], and direct conversion of fibroblasts into an alternate fate has also been facilitated by forced expression of lineage-specific TFs [6,7,8,9,10,11]. Cancer cells are plastic, meaning they have the ability to generate an alternate fate by rewiring their transcriptional circuitry [12], suggesting that cancer cells have the capacity to generate a benign cell fate by activating lineage-specific transcriptional networks. Along this line, cancer cells can be reprogrammed to an iPSC-like state [13,14,15,16], cancer stem cell (CSC) [15,17,18,19,20], or benign fate [21,22,23] following forced expression of TFs.

To decipher the molecular mechanisms that drive malignancy in a cell- and tissue-specific manner, it is crucial to understand the dominant oncogenic TFs that distinguish cancer cells from normal cells. As oncogenic transformation typically involves aberrant activation of primitive developmental programs, a process that is mirrored during the acquisition of iPS cells, it is plausible that tools that faithfully identify cell-type defining TFs can be successfully applied to reverse engineer cancer cells to a benign or drug-sensitive fate. Identifying GRNs across various types of cancer can aid in understanding oncogenic mechanisms and serve as targets for cancer therapy, making it highly relevant in the field of oncology. Despite a compendium of studies which have generated expression datasets for a variety of cancer types, GRNs that define cancer types have not been fully elucidated.

Here, we utilized the core engine of a network biology platform [24] to identify cancer type-specific GRNs for 17 types of cancers (adrenal, breast, cervical, esophageal, colon, lung, brain/glioma, leukemia, lymphoid, melanoma, pancreatic, prostate, stomach/gastric, thyroid, uterine, and uveal). The choice of these specific types was driven by the availability of gene expression data from tumor samples in public databases. Given the requirement for a sufficient volume of sample data to conduct a reliable analysis, we grouped all subtypes of each cancer together. This aggregation approach increases the pool of available data, thereby enhancing the statistical power and reliability of the analysis. However, it is important to recognize that this method may mask some subtype-specific characteristics.

The resulting platform, which we generated using a compendium of publicly available cancer expression datasets, is a robust tool to identify cancer type-specific GRNs, which are comprised of multiple TFs. We describe the performance of the platform and identify core TFs and GRNs for multiple cancer types. We also compared normal tissue and cell types with cancer type-specific networks to identify candidate oncogenic reprogramming factors, candidate therapeutic targets, or biomarkers. Findings from this study also show that the expression of network-influencing TFs can predict the survival of cancer patients. Overall, these findings provide a resource to study cancer-specific transcriptional networks.

## 2. Materials and Methods

### 2.1. Training the Cancer Cellnet Model

1. *Generation of metadata file (training sample table)*

To obtain the full training datasets, GEO was used to search for expression profiles of 17 types of cancer (Tables S1 and S2) using the Affymetrix HG133 plus 2 platform. Raw *.CEL files containing expression data from cancer samples were used (Table S2). A csv file or R data frame was created that holds the metadata for each expression sample as previously described [24,25,26]. The metadata includes a distinct sample identifier, the name of the raw data file, and an annotation (experimental group). Table S2 is the csv version of the training metatable file.

2. *Pre-processing of training data*

Pre-processing was performed using CellNet as previously described [24,25,26]. Pre-processing consists of retrieving the raw expression files, extracting them, and then normalizing them. Affymetrix microarrays that failed the quality control check were excluded from training CellNet. The raw CEL files were processed to correct for background and summarized as probeset values. The values of probesets mapping to the same gene were then averaged. To normalize each array, the gene expression values were divided by the total gene expression per array. Samples were then selected for GRN reconstruction. See https://github.com/KidderLab/CancerTFs (accessed on 10 August 2023) for additional training details. The pre-processing of both the training data and the query data to be analyzed by CellNet was performed using the same methodology.

3. *GRN construction, training, and validation:*

This step generated cancer type-specific gene regulatory networks (GRNs). The evaluation of classifiers developed through GRN construction takes place in this step. The process involves dividing the training data equally into two sections. One part is employed to train the CellNet model, while the other part serves as validation data. The generated results enable the construction of heat maps and precision–recall curves, thereby facilitating the assessment of the classifiers.

Using CellNet on the processed data, we generated several outputs: first, the classification score, which provides insight into the extent to which the expression profiles of the query samples resemble each of the reference cancer/tumor types. Secondly, the GRN Status, which serves as a metric to assess the level of establishment of cancer/tumor-specific GRN within the sample. Finally, a ranked list of transcription factors (TFs), whose expression modulation has the greatest probability of driving the desired fate change, referred to as the network influence score (NIS).

4. *Precision recall curves*

The performance of the classification model was evaluated using a precision versus sensitivity plot, where precision represents the fraction of correctly classified positive samples, and sensitivity represents the fraction of all positive samples that were correctly classified for a specific cancer type, with each point on the plot representing the classification score at which these metrics were calculated. Precision and sensitivity were calculated using the number of true positive calls divided by the number of positive calls and the sum of true positives and false negatives, respectively.

### 2.2. Querying Normal Tissue Using Training Cancer Data

To obtain query datasets to compare to the trained cancer data, GEO was used to search for expression profiles of normal tissues (Tables S3 and S4) using the Affymetrix HG133 plus 2 platform. Raw *.CEL files containing expression data from normal samples were used (Tables S3 and S4). A csv file or R data frame was created that holds the metadata for each expression sample as described above. The metadata included a distinct sample identifier, the name of the raw data file, and an annotation (experimental group). Table S4 is the csv version of the query metatable file. This file served as a means to query samples in the CellNet output. Normal cells (query) were utilized for comparison with cancer cells (target). In a similar manner to training data, raw data from normal cells were extracted, and the expression data were normalized.

### 2.3. Gene Ontology Functional Annotation

Enrichr [27], which features integrated gene-set libraries, was used to functionally annotate network-influencing genes. ChEA [28] 2022 Enrichr analyzes expression data through gene-list enrichment analysis using a database of ChIP-chip, ChIP-seq, and ChIP-PET. ChEA calculates the over-representation of transcription factor targets from the ChEA database from an input list of genes. Enrichr was used to generate clustergrams of TF enrichment and the umap scatterplot of the ChEA 2022 gene set library.

### 2.4. Kaplan–Meier Plots

KM-plotter [29,30], the web-based survival analysis tool, was used to assess clinical outcomes, including survival of cancer patients with high and low expression of network influencing score TFs. KM-plotter for pan-cancer data [29] was used to investigate the correlation between gene expression and survival rates in patients with breast, cervical, esophageal, lung, stomach, and uterine cancer, while the KM-plotter for colon cancer [30] was used to analyze the link between expression and patient survival in colon cancer cases.

The survival curves partition patient outcomes into two groups based on low (black) and high (red) gene expression levels. Cox proportional hazards regression analyses were used to study the link between gene expression and overall survival. KM-plotter [29,30] was utilized to generate log-rank *p*-values. The differential survival rates were visualized using Kaplan–Meier survival plots. We used the KM-plotter’s auto-select best cutoff feature, which calculates all possible cutoffs between the lower and upper expression quartiles. Each cutoff was independently assessed using Cox regression. We adjusted for multiple hypotheses testing using the false discovery rate (FDR), accepting only results with an FDR of less than 10%. The cutoff with the smallest *p*-value was employed for the final Kaplan–Meier plot.

### 2.5. Cancer Dependency Analysis Using the Depmap Database

The cancer dependency database [31] was used to explore the essential roles of genes within a prostate cancer GRN identified in this study. The depmap portal was used to acquire dependency scores (CERES) for prostate cancer subnetwork genes.

## 3. Results

### 3.1. Application of a Network Biology Platform to Identify Cancer Type-Specific Gene Regulatory Networks

**Figure 1:**
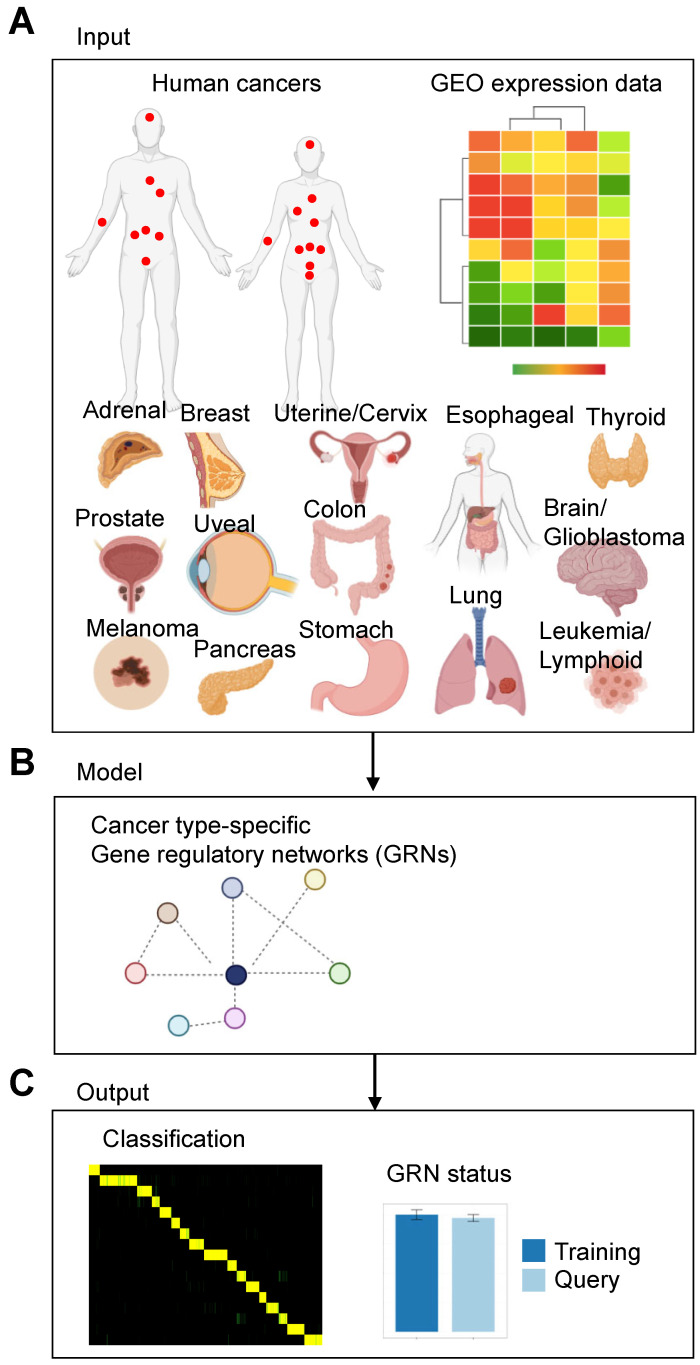
Schematic of the model to identify gene regulatory networks (GRNs) in cancer. (**A**) We adapted the CellNet model to identify GRNs in 17 types of human cancer. (**B**) Gene expression data were used to train a random forest classifier to reconstruct GRNs. (**C**) The CellNet model produces several outputs [25]: (1) a score indicating how closely a query sample’s expression profile matches each reference cancer type. (2) A measure of the degree to which a GRN is established in a query sample. (3) A ranking of transcription factors based on the likelihood that altering their expression would enhance the desired fate change (network influence score).

We utilized a computational biology platform [24], which uses random forest classification to evaluate the similarity of transcriptional profiles between in vivo cells and engineered or differentiated in vitro cells. Here, we used a similar strategy to construct a platform to identify cancer type-specific GRN for 17 types of human cancer (Figure 1A). Gene expression has been successfully used to reconstruct GRNs [32,33], and CellNet deploys an InfoMap community detection algorithm [34] to gain insight into subnetworks [25] and to assign subnetworks to specific cell and tissue types. GRNs regulate distinct expression programs of a given cell type and control cell type-specific response to extrinsic and intrinsic signals. GRNs control cell type identity by regulating distinct expression programs [35]. We used public gene expression data from 17 solid and liquid tumor types (Figure 1A; Tables S1 and S2) to train a random forest classifier (Figure 1B,C).

**Figure 2:**
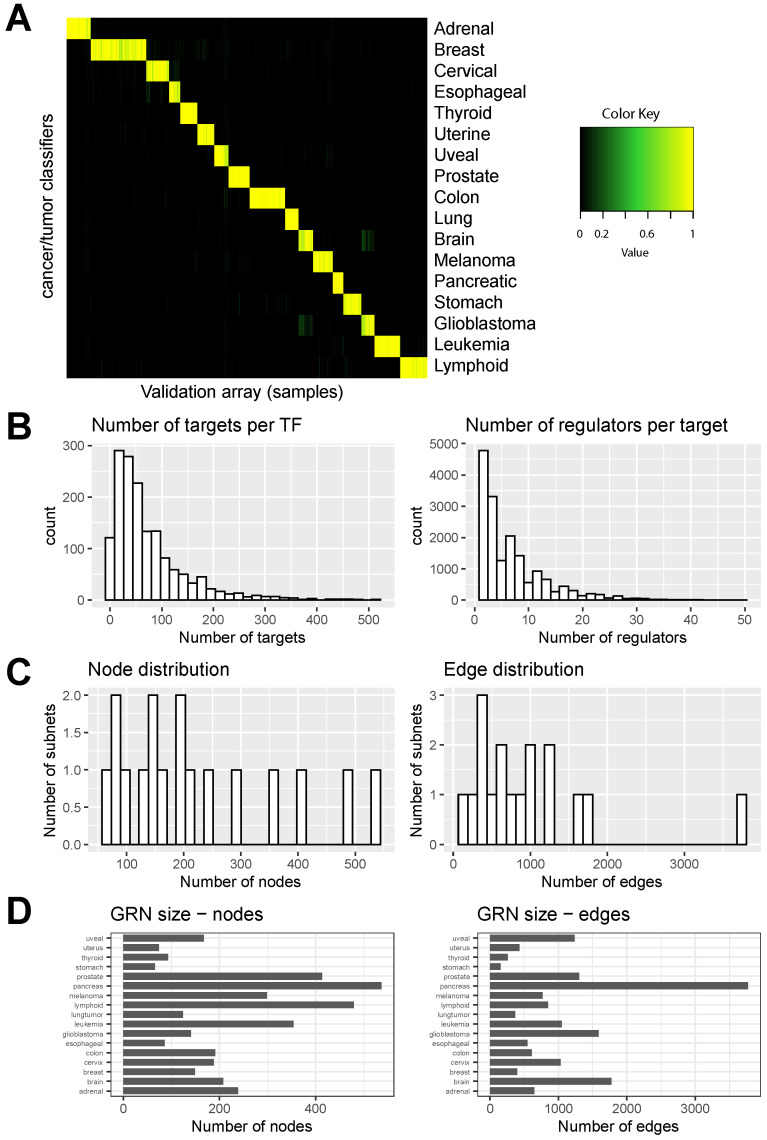
Validation of the CellNet model applied to pan-cancer expression data. (**A**) Heatmap showing the classification of a human pan-cancer validation dataset. Classifiers were created for each cancer and tumor type. Rows show cancer/tumor classifiers; columns show validation arrays (samples). High classification scores suggest a high probability of query samples expressing GRN genes at a level similar to the training data’s cancer/tumor type. (**B**) The number of targets per transcription factor (TF) and the number of regulators per target. (**C**) The node distribution and edge distribution (number of nodes and edges). (**D**) GRN size. Number of nodes and edges for each cancer/tumor type is shown in the barplot.

Figure 2A illustrates the performance of the classifier through a heatmap. In this heatmap, each column represents an input sample, while each row corresponds to a different classifier. The color intensity depicts the probability of a given input sample being assigned to a particular class based on the classifiers. A clear pattern or clustering within the heatmap indicates a high degree of agreement between classifiers and successful discrimination between different classes based on the input samples. The diagonal pattern in Figure 2A shows the indicated classification results. The GRN performance also describes a prediction of the number of gene targets per TF (Figure 2B, left; Table S5) and the number of regulators per target gene (Figure 2B, right; Table S6).

Genes whose promoters are bound by TF were derived from the gold standard ChIP-Seq data from the ENCODE project [36], the Escape database [37] containing ChIP-Seq data from TFs bound to gene promoters, and genes that are differentially expressed upon forced expression [38]. Integration of these three gold-standard datasets was previously used to predict TF–target gene interactions [24,25]. Identifying GRN components that are cell or tissue-specific requires characterizing genes (nodes) that interact with one another in specific subnetworks relative to nodes in other networks. The distribution of nodes (genes) across subnets and edges (link) is shown in Figure 2C. In addition, varying GRN sizes for 17 types of cancer highlight the complexity of cancer-specific networks, as shown in Figure 2D.

**Figure 3:**
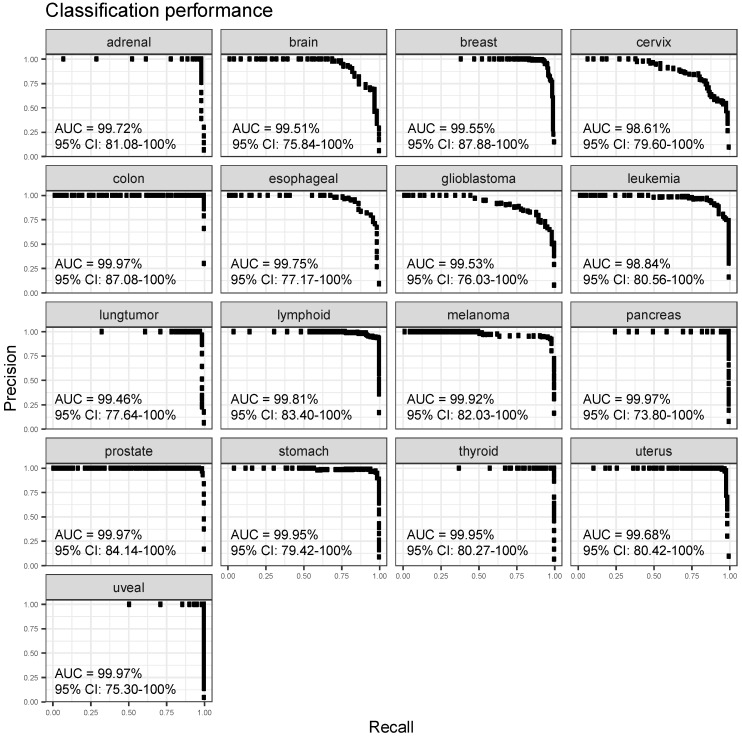
Precision recall curves for each cancer/tumor expression classifier. The x-axis represents the sensitivity/recall or fraction of samples from cancer/tumor type that are classified as such. The precision on the y-axis refers to the proportion of true positives (correctly classified samples) among all samples classified as positive for specific cancer or tumor type. Each dot displays precision and sensitivity at a classification score cutoff. As the cutoff rises, precision increases and recall decreases.

The classifier performance was assessed using precision sensitivity curves for each gene expression cancer type classifier as previously described [24,25] (Figure 3). The mean AUC surpassed 98% for all 17 cancer types, with all 95% confidence intervals ranging from 73% to 100%.

**Figure 4:**
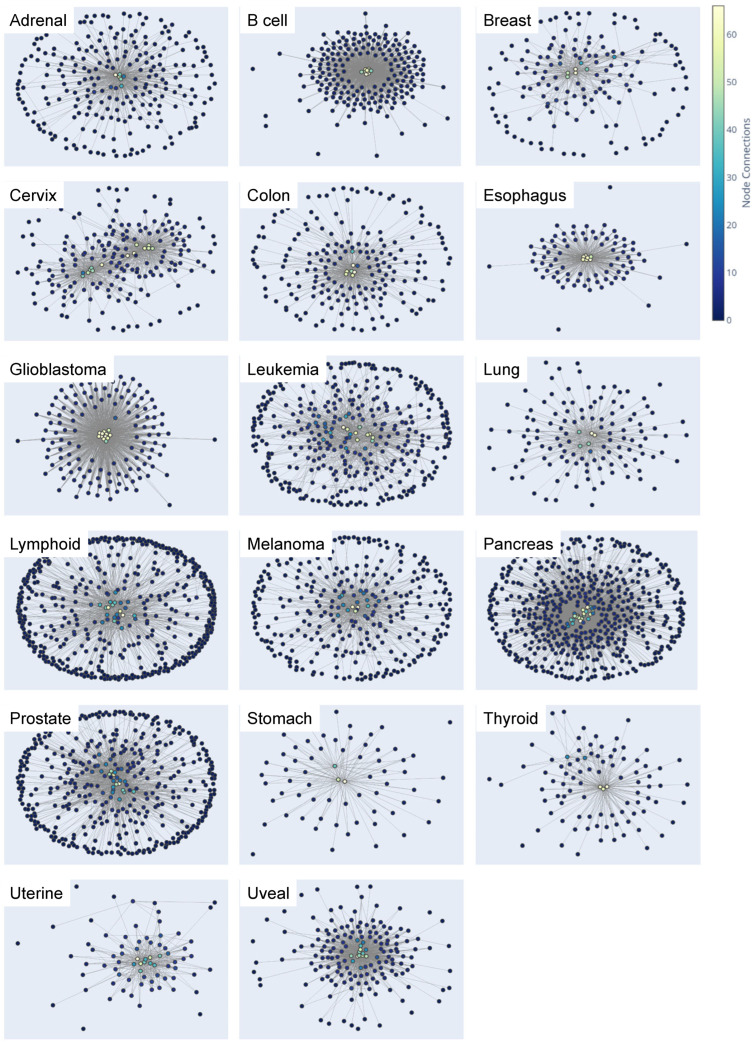
Visualization of cancer type-specific gene regulatory networks. This visualization illustrates the structural characteristics of cancer type-specific GRNs. Nodes in the network represent genes, and edges represent regulatory interactions between them. Node color is proportional to the network influence of the gene (number of node connections), and nodes with higher weights are grouped together, indicating potential cancer type-specific TF hubs. These TF hubs may comprise core regulatory networks of genes with elevated expression in a cancer type-specific manner. The visualization was generated using the Python libraries networkx and plotly. Detailed information on the GRNs can be found in Table S8.

We also assessed the cancer type-specific networks’ structural characteristics, including each GRN’s node and edge counts (Table S7), their specific nodes and edges (Figure 4), and cancer type-specific GRNs (Table S8). The visualizations indicate that connections (edges) with higher weights form cancer type-specific TF hubs (Figure 4) comprising interactions among core TFs and reveal GRNs consisting of genes with elevated expression in a cancer type-specific manner. Hubs with light blue, green, and yellow nodes signify more connections between core cancer type-specific TFs, highlighting potential therapeutic targets or biomarkers.

### 3.2. Exploration and Analysis of Network Influencing Genes

**Figure 5:**
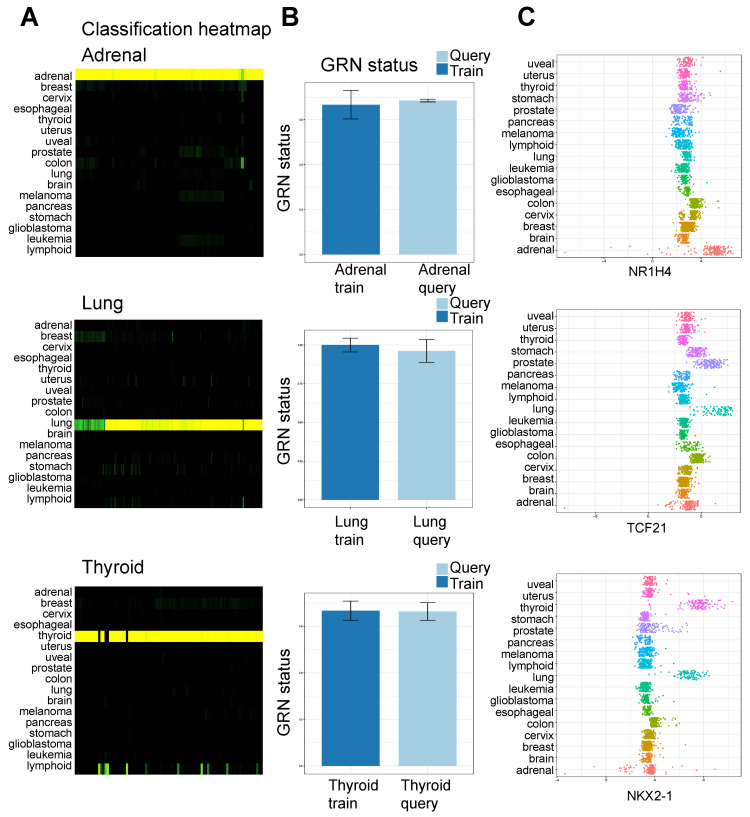
Analysis of pan-cancer expression data. (**A**) Classification heatmap of the normal query data relative to trained cancer data. Rows indicate cancer/tumor type of the training data, and columns are normal query samples. Each square is colored according to the classification score of the query sample for a particular cancer/tumor type (score: 0 to 1). The yellow color in the classification heatmaps indicates the predicted assignment of a sample to a specific cell type based on its gene expression profile. The shade of yellow corresponds to the degree of confidence in the assignment, with brighter shades of yellow indicating higher confidence in the classification. The yellow color in the heatmap is used to visualize the predicted cell type assignments for each. (**B**) Cancer/tumor type GRN status of normal adrenal, lung, and thyroid tissue relative to cancer/tumor samples. GRN status shows the degree to which cell/tissue GRN is established in the training (dark blue) and query (light blue) samples. The GRN status is calculated as the average z-score of all genes in a cell/tissue GRN, weighted by their significance to the related cell/tissue classifier. The GRN status is standardized to the average GRN status of the training data samples for the particular cell/tissue type [24,25]. Error bars represent mean ± 1 s.d. (**C**) Expression of cancer/tumor-specific genes. Scatter plots show the expression of adrenal, lung, and thyroid-specific genes across the pan-cancer expression datasets. Each point depicts the expression of a gene in an individual training dataset. Cancer/tumor types are represented by different colors. X-axis: expression level; y-axis: cancer/tumor type.

The dataset that was employed to train CellNet and create the classifiers consists of expression data collected from primary tumor samples that come from 17 different types of cancer. Normal cell and tissue types were used as query datasets to assess the expression differences between cancer and normal tissues. We applied this classification strategy to identify key TF regulators that distinguish normal cells from cancer cells. Example classifications include normal adrenal, lung, and thyroid tissue classified as adrenal, lung, and thyroid, respectively, and reached >90% GRN status (Figure 5A), and normal B cells classified as leukemia (Figure S1) and reached 98% leukemia GRN status (Figure S2). Figure 5A and Figure S1 show classification heatmaps of all cancer types.

The barplots in Figure 5B and Figure S2 show the degree to which the cancer cell GRN is established in each of the query samples (e.g., adrenal, lung, thyroid). The GRN status barplot compares the predicted regulatory networks for a query dataset to the known regulatory networks in the training dataset (Figure 5B and Table S9). The plot provides a summary of the similarity between the predicted regulatory networks for the query dataset and the training dataset.

Additionally, neurons are classified as brain cancer or glioblastoma, and the uterus is classified as uterine cancer. Uterine tissue is comprised of various cell types, such as endometrial tissue cells (luminal and glandular epithelium), fibroblasts/stromal cells, smooth muscle and endothelial cells, and immune cells. As such, the uterine tissue cellular composition partially overlaps other organs resulting in a slightly lower GRN status (93%). When we applied this strategy to a variety of normal equivalent cells, we found that they were largely classified as their tumor counterpart (Figure S1) and reached a high GRN establishment score. Representative examples of genes whose expression is enriched in cancer-related to normal tissue (top gene for each cancer type) in the full training data set are shown in Figure 5C and Figure S3. The plots display genes with elevated expression in specific cancer types compared to others.

Network influencing genes within GRNs that distinguish normal cells from their cancerous counterparts (cancer type-specific GRNs; Table S10) were further investigated. Gene expression data from normal cells (query cell type; Table S4) were compared to the GRN of cancer cells (trained data). The study identified distinct counts of TFs across various cancer types relative to normal tissue.

### 3.3. Functional Annotation of Network Influencing Genes

**Figure 6:**
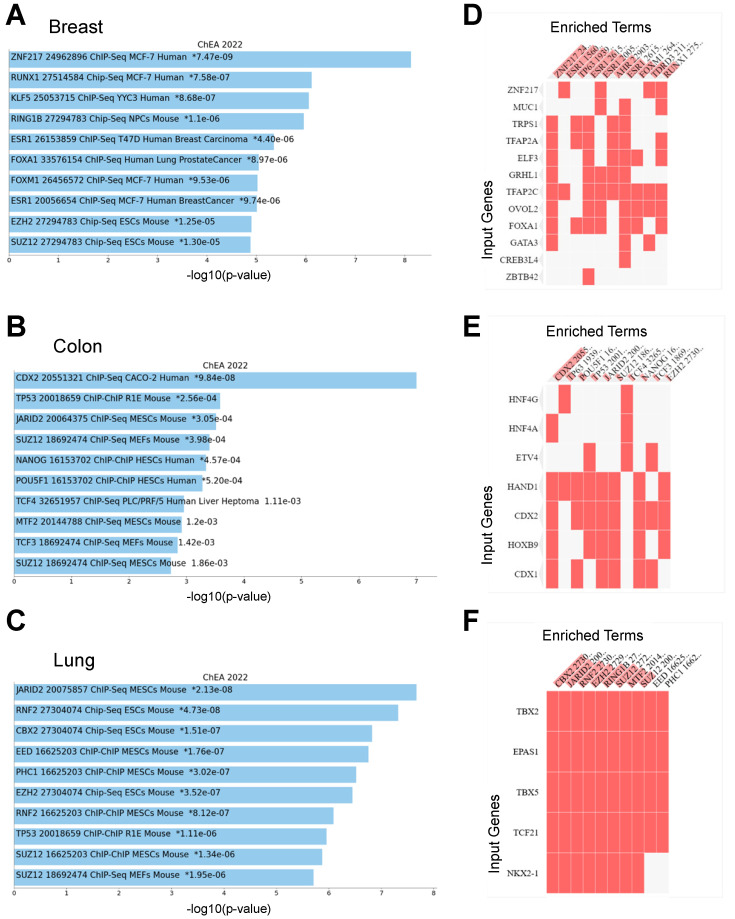
Target gene transcription factor associations of cancer/tumor type-specific genes. Enrichr ChEA target gene TF associations of cancer/tumor specific genes identified by querying normal cell/tissue expression data to trained cancer/tumor expression data. Results from Enrichr ChEA analysis for breast, colon, and lung cancer/tumor-specific genes: (**A**–**C**) Bar plots showing the top 10 enriched terms from the ChEA_2022 gene set library. Plots are ordered by −log10 (*p*-value) to display enrichment significance, with the actual *p*-value provided alongside. The top-most term signifies the highest overlap with the queried gene set. The number next to the asterix indicates the −log10 (*p*-value). (**D**–**F**) Clustergrams depict enriched terms as columns (transcription factor binding targets obtained from public ChIP-Seq datasets) and input genes as rows (cancer type-specific genes identified in this study). The intersection points in the matrix denote an association between a particular gene and a term.

Enrichr [27] was used to identify significantly enriched terms identified by comparing gene expression data from normal cells to cancer cells. This method aids in validating the detection of established cancer-related genes within the dataset. UMAP visualization for the ‘ChEA 2022’ analysis of cancer type-specific GRNs showed enrichment for known TF binding profiles (Figures S4 and S5; Table S11). The 10 most enriched terms relevant to the gene set ranked according to the -log10(*p*-value) are shown in Figure 6A–C and Figure S6. As an example, our results showed enrichment of RUNX1, ESR1, FOXA1, and FOXM1 target genes in breast cancer type-specific GRNs (Figure 6A). Our results also show enrichment of CDX2, TP53, JARID2, SUZ12, NANOG, OCT4, and TCF3 targets in colon cancer relative to normal cells (Figure 6B).

A detailed examination of all studied cancer types is presented in Figure S6. In addition, the Enrichr ChEA 2022 clustergrams reveal enriched terms (ChIP-Seq binding of TFs) for genes identified in cancer-specific GRNs (Figure 6D–F and Figure S7).

### 3.4. Implications of Elevated Gene Expression on Survival Rates of Cancer Patients

**Figure 7:**
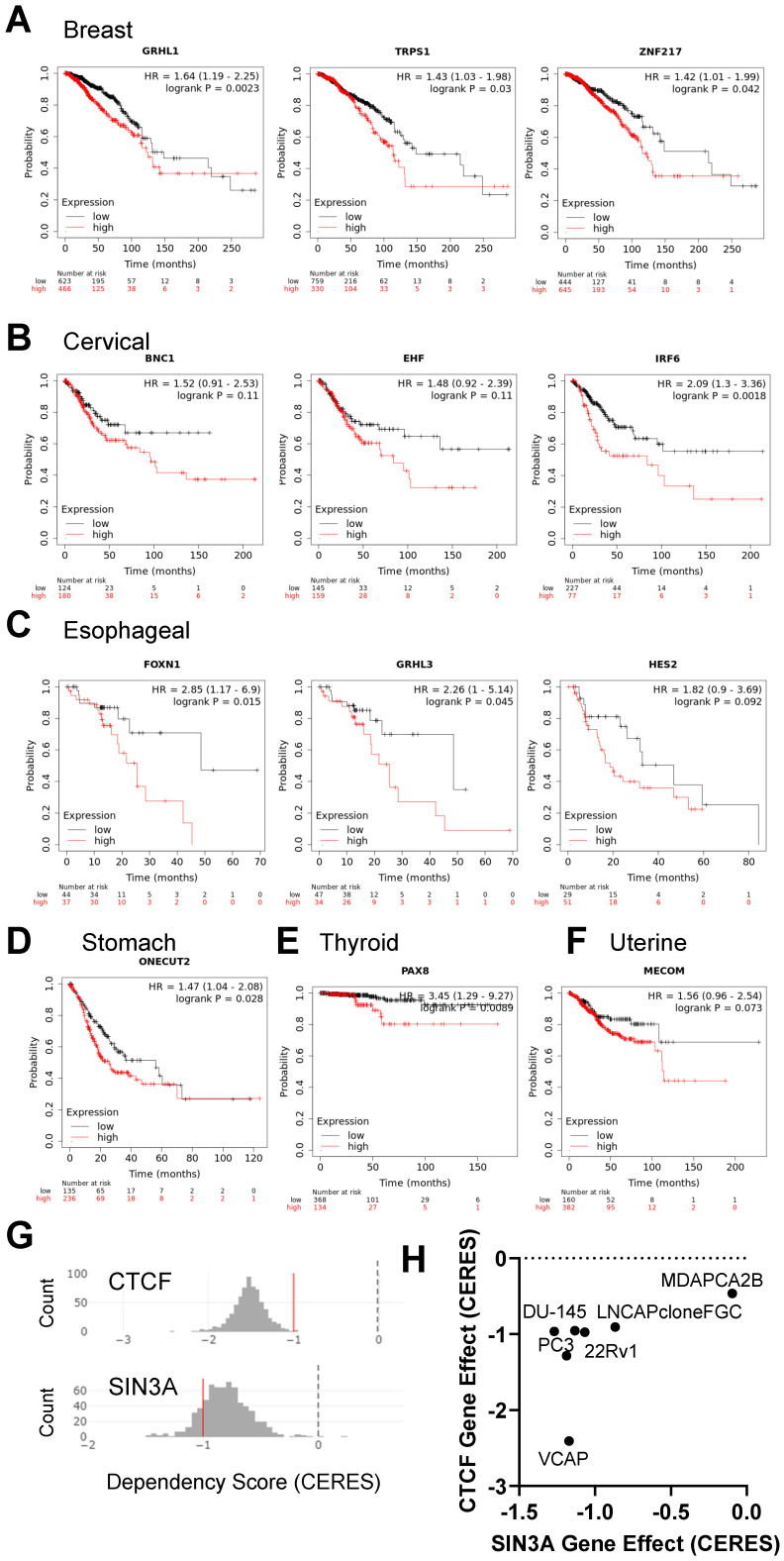
Association between expression of cancer-specific genes and survival. Kaplan–Meier curves for the overall survival of cancer patients exhibiting high (red) or low (black) expression of cancer/tumor specific genes in (**A**) breast, (**B**) cervical, (**C**) esophogeal, (**D**) stomach, (**E**) thyroid, and (**F**) uterine cancer. Patients are split by low and high expression (see methods for Kaplan–Meier plots). (**G**,**H**) Cancer dependency scores (gene effect, CERES) for CTCF and SIN3A in prostate cancer cell lines. (**G**) Histogram plot distribution of dependency scores across all cancer cell lines evaluated in the CRISPR screen [31]. A low negative dependency score indicates a higher likelihood that a gene is essential for the cell’s survival. A depmap score of less than −1 is highly essential to a cell. (**H**) Scatter plot distribution of CERES scores. Essential genes have a negative CERES score.

A further exploration of genes enriched in cancer cells relative to normal cells (Table S10) using Kaplan–Meier Plotter [29,30] revealed that breast cancer patients with elevated expression of GRHL1, TRPS1, and ZHF217 have a decreased rate of survival relative to patients with low expression of these genes (Figure 7A, Table S12). Additionally, cervical cancer patients with elevated expression of BNC1, EHF, and IRF6 have a decreased rate of survival relative to patients with low expression of these genes (Figure 7B). Esophageal cancer patients with high expression of FOXN1, GRHL3, and HES2 (Figure 7C), stomach cancer patients with high expression of ONECUT2 (Figure 7D), thyroid cancer patients with high expression of PAX8 (Figure 7E), and uterine cancer patients that expressed elevated levels of MECOM (Figure 7F) had decreased survival. Moreover, lung cancer patients with elevated expression of TCF21, NKX2-1, TBX2, and TBX5 exhibited a decreased rate of survival relative to patients with low gene expression (Figure S8A). We also found that pancreatic cancer patients with elevated expression of MAPK1, LRRFIP1, SP110, and NFE2 had a decreased rate of survival relative to patients with lower expression of these genes (Figure S8B). Our findings demonstrate the potential of GRNs in identifying genes that may be associated with important clinical outcomes, including patient survival.

To gain further insight into GRNs of prostate cancer, candidate core transcriptional regulatory factors were selected based on protein–protein interactions within the GRN [39] with elevated expression in prostate cancer and their cancer dependency score in prostate cancer cells [31]. An exploration of the prostate cancer GRN revealed a subnetwork comprised of two chromatin constituents, CTCF and SIN3A (Figure 7G,H). Both CTCF and SIN3A were observed to have overexpression in prostate cancer compared to normal tissue. Their low depmap dependency scores suggest these genes are crucial for the proliferation of prostate cancer cells. Both CTCF and SIN3A were found to have low depmap dependency scores for multiple prostate cancer cell lines (Figure 7H).

SIN3A, which is a scaffolding protein associated with the nucleosome remodeling domain complex [40], and CTCF, a known chromatin insulator and regulator of long-range chromatin interactions, were found to be key components of this subnetwork, suggesting their potential importance in regulating gene expression in prostate cancer cells.

## 4. Discussion

Here, we implemented an algorithmic platform [24] to identify (1) cancer type-specific GRNs and (2) TFs that distinguish cancer cells from normal cells. Our algorithmic identification of regulatory nodes (genes) for multiple types of cancers describes distinct differences in the expression of TFs between cancer cells and normal cells.

TFs regulate cell identity and may be useful in engineering cell models of transformation and tumor formation. We propose that identification of genes whose expression is enriched in cancer cells relative to normal cells may serve as biomarkers of tumorigenesis or potential therapeutic targets. While predicting the expression of genes within GRNs is challenging, our approach simplifies complex networks to prioritize key TFs that regulate cell identity. The study also aimed to understand how GRNs differ between normal cells and cancer cells, as these differences can provide valuable information about oncogenic networks that drive tumorigenesis. By conducting a comparative analysis of GRNs between normal and tumor cells, this study identified distinct gene expression patterns. Our results also provide a rich resource to explore and understand expression patterns that exist in a wide range of cancer types. By mining cancer type-specific GRNs across a broad range of cancer subtypes, it may be possible to identify common patterns and mechanisms of tumor formation, which could lead to the development of more effective treatments for cancer.

### 4.1. Performance Evaluation and Insights Derived from the Classifier and Gene Regulatory Networks

The study utilized a classification strategy to identify cancer type-specific GRNs and key TFs that distinguish normal cells and cancer cells. By using normal cell gene expression data as the query and contrasting it with GRNs generated from cancer cells, we were able to identify network-influencing genes within cancer GRNs.

The examination of differentially expressed genes between normal and cancer cells and the structural characteristics of these cancer type-specific GRNs may point towards potential therapeutic targets or biomarkers. These alterations could potentially play a critical role in disease onset or progression. Targeting these hubs could hinder cancer progression and improve treatment outcomes by disrupting oncogenic pathways or serving as models for oncogenic transformation in various cancer types. These findings lay the groundwork for exploring how targeting these may impede cancer progression and improve treatment outcomes.

### 4.2. Network Influencing Genes within Cancer Gene Regulatory Networks

Here, we conducted a comprehensive exploration and analysis of network-influencing genes within cancer type-specific GRNs, contrasting the gene expression data of normal cells with the GRN of various cancer cells. Our results revealed distinct TFs in different cancer types, providing insights into the gene regulatory alterations in cancer cells. Using Enrichr [27] and UMAP visualization, the study further validated the presence of established cancer-related genes and identified enrichments for known TF binding profiles. As an example, FOXA1 was found enriched in breast cancer type-specific GRNs, and several target genes were identified in colon cancer relative to normal cells. These findings establish the foundation for understanding the specific roles of these genes in breast cancer and their potential influence on disease prognosis and recurrence. FOXA1, a master transcriptional regulator, has been found to be overexpressed in luminal A and luminal B breast cancer subtypes, indicating its significant role in subtype-specific gene expression patterns [41]. We also identified genes that are expressed at higher levels in colon cancer relative to the normal colon, such as CDX2. The intestinal restricted transcription factor CDX2 has been implicated in colon cancer, where it functions as a tumor suppressor [42]. These findings provide key insights into the expression of TFs in normal cells relative to cancer cells.

### 4.3. Gene Regulatory Networks in Prostate Cancer

In this study, further exploration of the GRNs of prostate cancer identified key transcriptional regulatory factors, CTCF and SIN3A. These two chromatin constituents exhibited overexpression in prostate cancer compared to normal tissue, and the low depmap dependency scores of these genes highlight their essential role in prostate cancer cell proliferation, suggesting their potential as crucial regulators of gene expression within prostate cancer cells. These findings set the stage for further exploration of the roles of CTCF and SIN3A in prostate cancer progression and resistance to treatment.

Expression of the chromatin insulator, CTCF, which is involved in transcriptional regulation and chromatin loop formation, is overexpressed in prostate cancer relative to normal prostate tissue [43]. Prostate cancer cells acquire de novo topologically-associated domains (TADs) enriched with CTCF binding relative to normal prostate tissue. These findings build on previous studies which demonstrated that prostate cancer cells acquire de novo TADs, enriched with CTCF binding relative to normal prostate tissue [44,45]. In addition, CRISPR-mediated deletion of prostate cancer risk-associated CTCF loop anchors resulted in the de-repression of gene expression [46]. Genome-wide association studies (GWAS) identified more than 100 prostate cancer risk loci [47,48,49,50,51], which were linked to long-range chromatin CTCF loop anchors which function to repress gene expression [46]. CTCF was also found to be a candidate prognostic biomarker for prostate cancer, and depletion of CTCF leads to reduced prostate cancer cell migration, invasion, and proliferation [43]. While CTCF is a known chromatin insulator, which blocks enhancer function between TADs, it is unclear how CTCF reprograms the epigenetic landscape of prostate cancer cells to drive prostate cancer tumor progression, castration-resistance, and enzalutamide-resistance.

### 4.4. Gene Regulatory Network Performance Measures

Our results also highlight the GRN performance measures of gene targets per TF and the number of regulators per target gene. Transcription factors with more targets are likely to have a more extensive regulatory role. In contrast, transcription factors with fewer targets can be associated with increased specificity. The number of targets per TF can have an impact on the performance of the platform, as it affects the accuracy of the predicted regulatory networks. The number of targets per TF is a trade-off between specificity and accuracy in predicting regulatory networks with the model, where TFs with more targets are likely to have a broader regulatory role but may pose challenges in predicting specific interactions, while TFs with fewer targets may be more specific but are harder to predict using gene expression data alone. Our results leverage insight from the complex interplay between transcription factors and their targets to develop GRN models for the identification of cancer type-specific genes. By unraveling oncogenic transcriptional networks across various cancer types, it underscores the potential of this approach in revealing critical transcriptional regulators.

Moreover, in our analysis, we discovered that different cancer types exhibited distinct numbers of nodes within their GRN (Figure 4; Table S8). This variation in the number of nodes, indicative of the complex regulatory landscape, highlights the uniqueness of each cancer type. The disparities in both the number of nodes and edges among various cancer types provide valuable insights into their specific genetic characteristics. These insights contribute to a deeper understanding of cancer-specific behaviors and may facilitate the development of more targeted therapeutic strategies.

## 5. Conclusions

In conclusion, this study presents GRN models for the identification of cancer type-specific genes. By providing a resource for investigating the oncogenic transcriptional networks across a wide spectrum of cancer types, this study underscores the potential of utilizing this approach to reveal critical transcriptional regulators involved in various cancers.

## Data Availability

The data presented in this study are available in this article (and Supplementary Materials). The corresponding code has been made publicly accessible and can be found at the following link: https://github.com/KidderLab/CancerTFs.

## Supplementary Materials

The following supporting information can be downloaded at: https://www.mdpi.com/article/10.3390/cancers15164167/s1. Figure S1. Classification of a human pan-cancer validation dataset. Heatmap illustrates the classification of the human pan-cancer validation dataset—rows display cancer/tumor classifiers, and columns represent validation arrays. High scores indicate a high likelihood of query samples expressing GRN genes similar to training data’s cancer/tumor type. Figure S2. Gene regulatory network status. Comparison of GRN status of normal tissue to cancer/tumor samples. GRN status reflects the level of cell/tissue GRN establishment in training (dark blue) and query (light blue) samples. GRN status is calculated as the average z-score of all genes, weighted by their significance to the related cell/tissue classifier. GRN status standardized to the average GRN status of training data for each cell/tissue type. Error bars represent mean ± 1 standard deviation. Figure S3. Expression of cancer type-specific genes. Expression of cancer/tumor-specific genes: scatter plots display adrenal, lung, and thyroid gene expression across pan-cancer datasets. Each point represents the expression of a gene in an individual training dataset, with different colors representing cancer/tumor types. X-axis displays the expression level, and the y-axis shows the cancer/tumor type. Figure S4. Scatterplot visualization for Enrichr ChEA 2022. Scatterplot of all terms in the ChEA_2022 gene set library for genes identified by comparing cancer cells with normal tissue. The plot shows terms in the library, each represented by a point. The gene set corresponding to each term was analyzed using TF-IDF, and the results were visualized through UMAP. Similar terms tend to be positioned closer together based on the first two dimensions of UMAP. The Leiden algorithm was used to group the terms into clusters, which are indicated by the color of the points. The size and darkness of a point indicate the level of enrichment for that term. Figure S5. Scatterplot visualization for Enrichr ChEA 2022. Scatterplot of all terms in the ChEA_2022 gene set library for genes identified by comparing cancer cells with normal tissue. The plot shows terms in the library, each represented by a point. The gene set corresponding to each term was analyzed using TF-IDF, and the results were visualized through UMAP. Similar terms tend to be positioned closer together based on the first two dimensions of UMAP. The Leiden algorithm was used to group the terms into clusters, which are indicated by the color of the points. The size and darkness of a point indicate the level of enrichment for that term. Figure S6. ENRICHR: ChEA 2022 Bar Chart. Results from Enrichr ChEA analysis: Bar plots showing the top 10 enriched terms from the ChEA_2022 gene set library. Plots are ordered by -log10 (*p*-value) to display enrichment significance, with the actual *p*-value provided alongside. The top-most term signifies the highest overlap with the queried gene set. Figure S7. ENRICHR: ChEA 2022 clustergram. Heatmap illustrating the association between cancer type-specific genes and enriched terms in the ChEA 2022 database. Analysis was performed using Enrichr. Columns show transcription factor binding targets obtained from public ChIP-Seq datasets, and the rows indicate the cancer type-specific genes identified in this study. The intersection points in the matrix denote an association between a particular gene and a term. Figure S8. Association between the expression of cancer-specific genes and survival. Kaplan–Meier curves for the overall survival of cancer patients exhibiting high (red) or low (black) expression of cancer/tumor-specific genes in (A) lung and (B) pancreatic cancer. Patients are split by low and high median expression; Table S1. Cancer types and sample count; Table S2. Metatable for cancer expression datasets; Table S3. Normal tissue samples used for queries; Table S4. Metatable for normal expression datasets; Table S5. Transcription factor targets; Table S6. Number of regulators per target; Table S7. GRN node and edge count; Table S8. Cancer Type-Specific GRNs; Table S9. Gene regulatory network status. Data related to Figure 5B and Figure S2; Table S10. GRN genes enriched in cancer cells relative to normal cells; Table S11. Adrenal cancer. ENRICHR: ChEA; Table S12. Kaplan-Meier Survival Analysis.

## References

[1] Takahashi K., Yamanaka S. (2006). Induction of pluripotent stem cells from mouse embryonic and adult fibroblast cultures by defined factors. Cell.

[2] Aasen T., Raya A., Barrero M.J., Garreta E., Consiglio A., Gonzalez F., Vassena R., Bilic J., Pekarik V., Tiscornia G. (2008). Efficient and rapid generation of induced pluripotent stem cells from human keratinocytes. Nat. Biotechnol..

[3] Loh Y.H., Hartung O., Li H., Guo C., Sahalie J.M., Manos P.D., Urbach A., Heffner G.C., Grskovic M., Vigneault F. (2010). Reprogramming of T cells from human peripheral blood. Cell Stem Cell.

[4] Staerk J., Dawlaty M.M., Gao Q., Maetzel D., Hanna J., Sommer C.A., Mostoslavsky G., Jaenisch R. (2010). Reprogramming of human peripheral blood cells to induced pluripotent stem cells. Cell Stem Cell.

[5] Kim J.B., Sebastiano V., Wu G., Arauzo-Bravo M.J., Sasse P., Gentile L., Ko K., Ruau D., Ehrich M., van den Boom D. (2009). Oct4-induced pluripotency in adult neural stem cells. Cell.

[6] Davis R.L., Weintraub H., Lassar A.B. (1987). Expression of a single transfected cDNA converts fibroblasts to myoblasts. Cell.

[7] Vierbuchen T., Ostermeier A., Pang Z.P., Kokubu Y., Sudhof T.C., Wernig M. (2010). Direct conversion of fibroblasts to functional neurons by defined factors. Nature.

[8] Ieda M., Fu J.D., Delgado-Olguin P., Vedantham V., Hayashi Y., Bruneau B.G., Srivastava D. (2010). Direct reprogramming of fibroblasts into functional cardiomyocytes by defined factors. Cell.

[9] Huang P., He Z., Ji S., Sun H., Xiang D., Liu C., Hu Y., Wang X., Hui L. (2011). Induction of functional hepatocyte-like cells from mouse fibroblasts by defined factors. Nature.

[10] Sekiya S., Suzuki A. (2011). Direct conversion of mouse fibroblasts to hepatocyte-like cells by defined factors. Nature.

[11] Szabo E., Rampalli S., Risueno R.M., Schnerch A., Mitchell R., Fiebig-Comyn A., Levadoux-Martin M., Bhatia M. (2010). Direct conversion of human fibroblasts to multilineage blood progenitors. Nature.

[12] Meacham C.E., Morrison S.J. (2013). Tumour heterogeneity and cancer cell plasticity. Nature.

[13] Utikal J., Maherali N., Kulalert W., Hochedlinger K. (2009). Sox2 is dispensable for the reprogramming of melanocytes and melanoma cells into induced pluripotent stem cells. J. Cell Sci..

[14] Miyoshi N., Ishii H., Nagai K., Hoshino H., Mimori K., Tanaka F., Nagano H., Sekimoto M., Doki Y., Mori M. (2010). Defined factors induce reprogramming of gastrointestinal cancer cells. Proc. Natl. Acad. Sci. USA.

[15] Carette J.E., Pruszak J., Varadarajan M., Blomen V.A., Gokhale S., Camargo F.D., Wernig M., Jaenisch R., Brummelkamp T.R. (2010). Generation of iPSCs from cultured human malignant cells. Blood.

[16] Saito S., Lin Y.C., Nakamura Y., Eckner R., Wuputra K., Kuo K.K., Lin C.S., Yokoyama K.K. (2019). Potential application of cell reprogramming techniques for cancer research. Cell Mol. Life Sci..

[17] Borges G.T., Vencio E.F., Vencio R.Z., Vessella R.L., Ware C.B., Liu A.Y. (2015). Reprogramming of prostate cancer cells—Technical challenges. Curr. Urol. Rep..

[18] Iskender B., Izgi K., Canatan H. (2016). Reprogramming bladder cancer cells for studying cancer initiation and progression. Tumour Biol..

[19] Corominas-Faja B., Cufi S., Oliveras-Ferraros C., Cuyas E., Lopez-Bonet E., Lupu R., Alarcon T., Vellon L., Iglesias J.M., Leis O. (2013). Nuclear reprogramming of luminal-like breast cancer cells generates Sox2-overexpressing cancer stem-like cellular states harboring transcriptional activation of the mTOR pathway. Cell Cycle.

[20] Kim J., Hoffman J.P., Alpaugh R.K., Rhim A.D., Reichert M., Stanger B.Z., Furth E.E., Sepulveda A.R., Yuan C.X., Won K.J. (2013). An iPSC line from human pancreatic ductal adenocarcinoma undergoes early to invasive stages of pancreatic cancer progression. Cell Rep..

[21] Zhang X., Cruz F.D., Terry M., Remotti F., Matushansky I. (2013). Terminal differentiation and loss of tumorigenicity of human cancers via pluripotency-based reprogramming. Oncogene.

[22] Mathieu J., Zhang Z., Zhou W., Wang A.J., Heddleston J.M., Pinna C.M., Hubaud A., Stadler B., Choi M., Bar M. (2011). HIF induces human embryonic stem cell markers in cancer cells. Cancer Res..

[23] Hoshino H., Nagano H., Haraguchi N., Nishikawa S., Tomokuni A., Kano Y., Fukusumi T., Saito T., Ozaki M., Sakai D. (2012). Hypoxia and TP53 deficiency for induced pluripotent stem cell-like properties in gastrointestinal cancer. Int. J. Oncol..

[24] Cahan P., Li H., Morris S.A., Lummertz da Rocha E., Daley G.Q., Collins J.J. (2014). CellNet: Network biology applied to stem cell engineering. Cell.

[25] Radley A.H., Schwab R.M., Tan Y., Kim J., Lo E.K.W., Cahan P. (2017). Assessment of engineered cells using CellNet and RNA-seq. Nat. Protoc..

[26] Morris S.A., Cahan P., Li H., Zhao A.M., San Roman A.K., Shivdasani R.A., Collins J.J., Daley G.Q. (2014). Dissecting engineered cell types and enhancing cell fate conversion via CellNet. Cell.

[27] Chen E.Y., Tan C.M., Kou Y., Duan Q., Wang Z., Meirelles G.V., Clark N.R., Ma’ayan A. (2013). Enrichr: Interactive and collaborative HTML5 gene list enrichment analysis tool. BMC Bioinform..

[28] Lachmann A., Xu H., Krishnan J., Berger S.I., Mazloom A.R., Ma’ayan A. (2010). ChEA: Transcription factor regulation inferred from integrating genome-wide ChIP-X experiments. Bioinformatics.

[29] Nagy A., Munkacsy G., Gyorffy B. (2021). Pancancer survival analysis of cancer hallmark genes. Sci. Rep..

[30] Lanczky A., Gyorffy B. (2021). Web-Based Survival Analysis Tool Tailored for Medical Research (KMplot): Development and Implementation. J. Med. Internet Res..

[31] Tsherniak A., Vazquez F., Montgomery P.G., Weir B.A., Kryukov G., Cowley G.S., Gill S., Harrington W.F., Pantel S., Krill-Burger J.M. (2017). Defining a Cancer Dependency Map. Cell.

[32] Margolin A.A., Wang K., Lim W.K., Kustagi M., Nemenman I., Califano A. (2006). Reverse engineering cellular networks. Nat. Protoc..

[33] Margolin A.A., Califano A. (2007). Theory and limitations of genetic network inference from microarray data. Ann. N. Y. Acad. Sci..

[34] Rosvall M., Bergstrom C.T. (2008). Maps of random walks on complex networks reveal community structure. Proc. Natl. Acad. Sci. USA.

[35] Davidson E.H., Erwin D.H. (2006). Gene regulatory networks and the evolution of animal body plans. Science.

[36] Mouse E.C., Stamatoyannopoulos J.A., Snyder M., Hardison R., Ren B., Gingeras T., Gilbert D.M., Groudine M., Bender M., Kaul R. (2012). An encyclopedia of mouse DNA elements (Mouse ENCODE). Genome Biol..

[37] Xu H., Baroukh C., Dannenfelser R., Chen E.Y., Tan C.M., Kou Y., Kim Y.E., Lemischka I.R., Ma’ayan A. (2013). ESCAPE: Database for integrating high-content published data collected from human and mouse embryonic stem cells. Database.

[38] Correa-Cerro L.S., Piao Y., Sharov A.A., Nishiyama A., Cadet J.S., Yu H., Sharova L.V., Xin L., Hoang H.G., Thomas M. (2011). Generation of mouse ES cell lines engineered for the forced induction of transcription factors. Sci. Rep..

[39] Szklarczyk D., Morris J.H., Cook H., Kuhn M., Wyder S., Simonovic M., Santos A., Doncheva N.T., Roth A., Bork P. (2017). The STRING database in 2017: Quality-controlled protein-protein association networks, made broadly accessible. Nucleic Acids Res..

[40] Jeanquartier F., Jean-Quartier C., Holzinger A. (2015). Integrated web visualizations for protein-protein interaction databases. BMC Bioinform..

[41] Li R., Campos J., Iida J. (2015). A Gene Regulatory Program in Human Breast Cancer. Genetics.

[42] Bonhomme C., Duluc I., Martin E., Chawengsaksophak K., Chenard M.P., Kedinger M., Beck F., Freund J.N., Domon-Dell C. (2003). The Cdx2 homeobox gene has a tumour suppressor function in the distal colon in addition to a homeotic role during gut development. Gut.

[43] Hoflmayer D., Steinhoff A., Hube-Magg C., Kluth M., Simon R., Burandt E., Tsourlakis M.C., Minner S., Sauter G., Buscheck F. (2020). Expression of CCCTC-binding factor (CTCF) is linked to poor prognosis in prostate cancer. Mol. Oncol..

[44] Taberlay P.C., Achinger-Kawecka J., Lun A.T., Buske F.A., Sabir K., Gould C.M., Zotenko E., Bert S.A., Giles K.A., Bauer D.C. (2016). Three-dimensional disorganization of the cancer genome occurs coincident with long-range genetic and epigenetic alterations. Genome Res..

[45] Rhie S.K., Perez A.A., Lay F.D., Schreiner S., Shi J., Polin J., Farnham P.J. (2019). A high-resolution 3D epigenomic map reveals insights into the creation of the prostate cancer transcriptome. Nat. Commun..

[46] Guo Y., Perez A.A., Hazelett D.J., Coetzee G.A., Rhie S.K., Farnham P.J. (2018). CRISPR-mediated deletion of prostate cancer risk-associated CTCF loop anchors identifies repressive chromatin loops. Genome Biol..

[47] Al Olama A.A., Kote-Jarai Z., Berndt S.I., Conti D.V., Schumacher F., Han Y., Benlloch S., Hazelett D.J., Wang Z., Saunders E. (2014). A meta-analysis of 87,040 individuals identifies 23 new susceptibility loci for prostate cancer. Nat. Genet..

[48] Thomas G., Jacobs K.B., Yeager M., Kraft P., Wacholder S., Orr N., Yu K., Chatterjee N., Welch R., Hutchinson A. (2008). Multiple loci identified in a genome-wide association study of prostate cancer. Nat. Genet..

[49] Eeles R.A., Kote-Jarai Z., Al Olama A.A., Giles G.G., Guy M., Severi G., Muir K., Hopper J.L., Henderson B.E., Haiman C.A. (2009). Identification of seven new prostate cancer susceptibility loci through a genome-wide association study. Nat. Genet..

[50] Berndt S.I., Wang Z., Yeager M., Alavanja M.C., Albanes D., Amundadottir L., Andriole G., Beane Freeman L., Campa D., Cancel-Tassin G. (2015). Two susceptibility loci identified for prostate cancer aggressiveness. Nat. Commun..

[51] Schumacher F.R., Al Olama A.A., Berndt S.I., Benlloch S., Ahmed M., Saunders E.J., Dadaev T., Leongamornlert D., Anokian E., Cieza-Borrella C. (2018). Association analyses of more than 140,000 men identify 63 new prostate cancer susceptibility loci. Nat. Genet..

