# Supplementary figures and images for: Identifying Cancer Type-Specific Transcriptional Programs through Network Analysis
Source: Cancers (Basel). 2023 Aug 18;15(16):4167. doi: 10.3390/cancers15164167 (PMC10453000; doi:10.3390/cancers15164167)

# Classification heatmap (query->target)

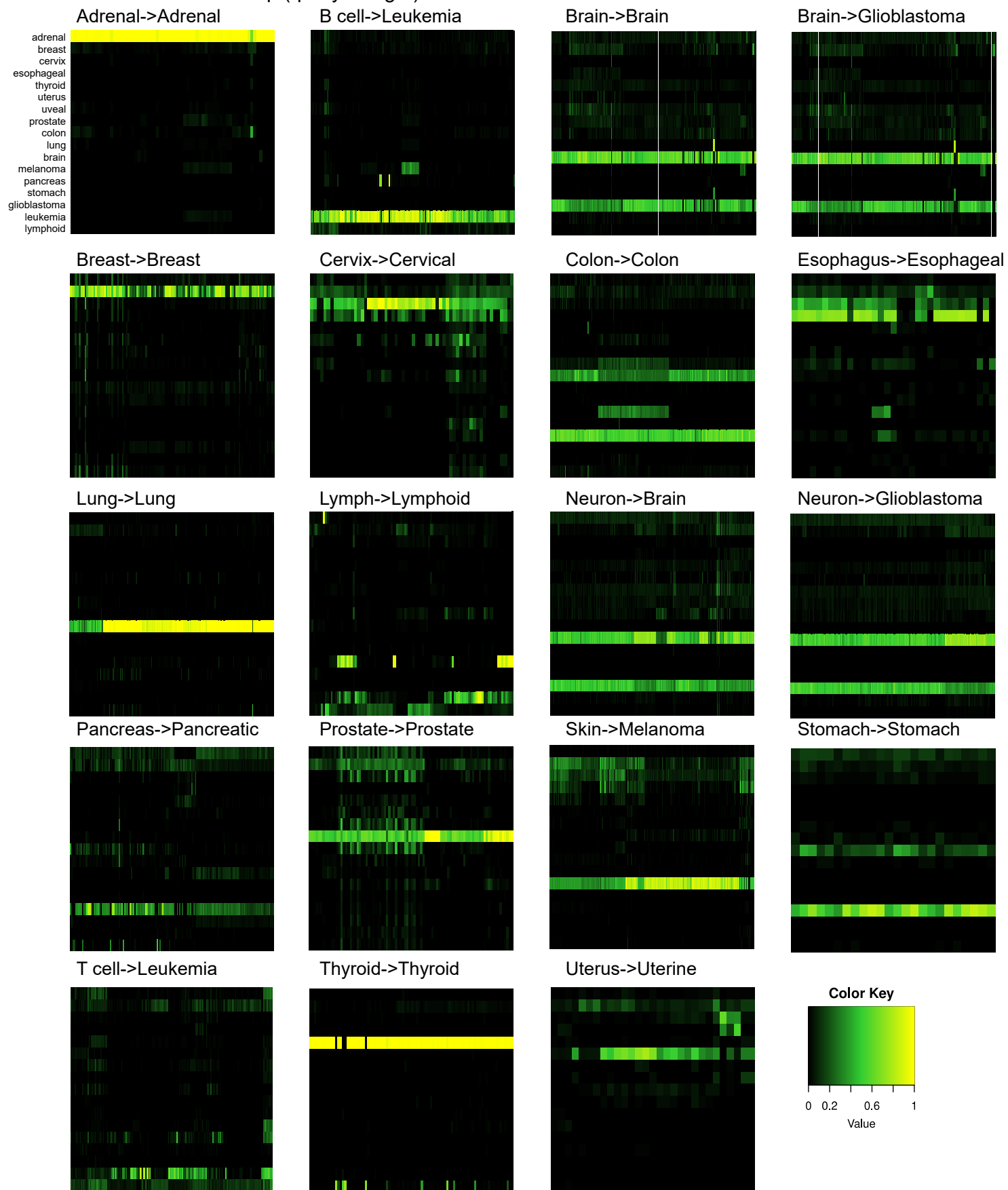

Figure S1

Supplement: Supplementary file 1 [file cancers-15-04167-s001.zip › FigureS1.pdf]

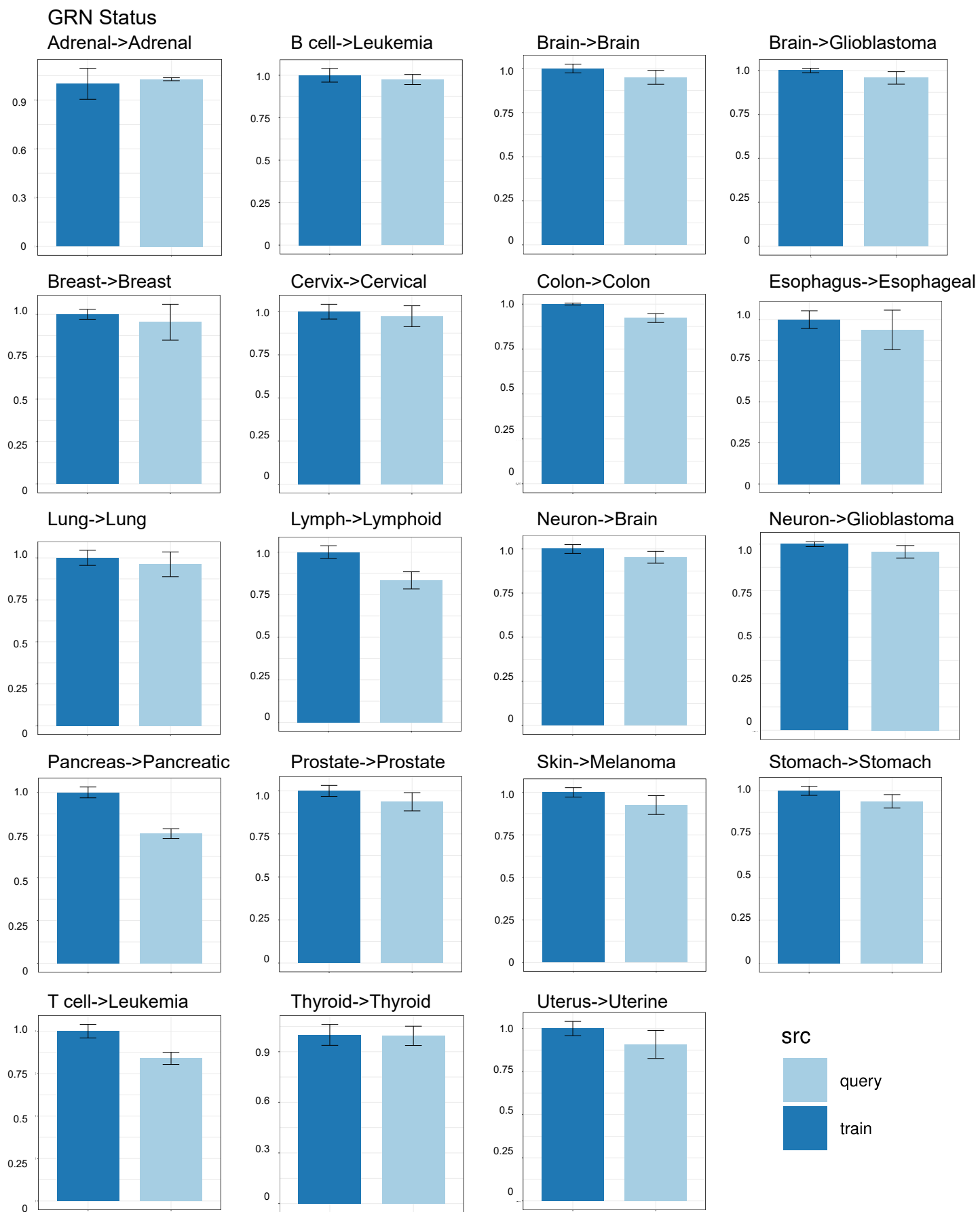

Figure S2

Supplement: Supplementary file 1 [file cancers-15-04167-s001.zip › FigureS2.pdf]

# GRN Status (guery->target)

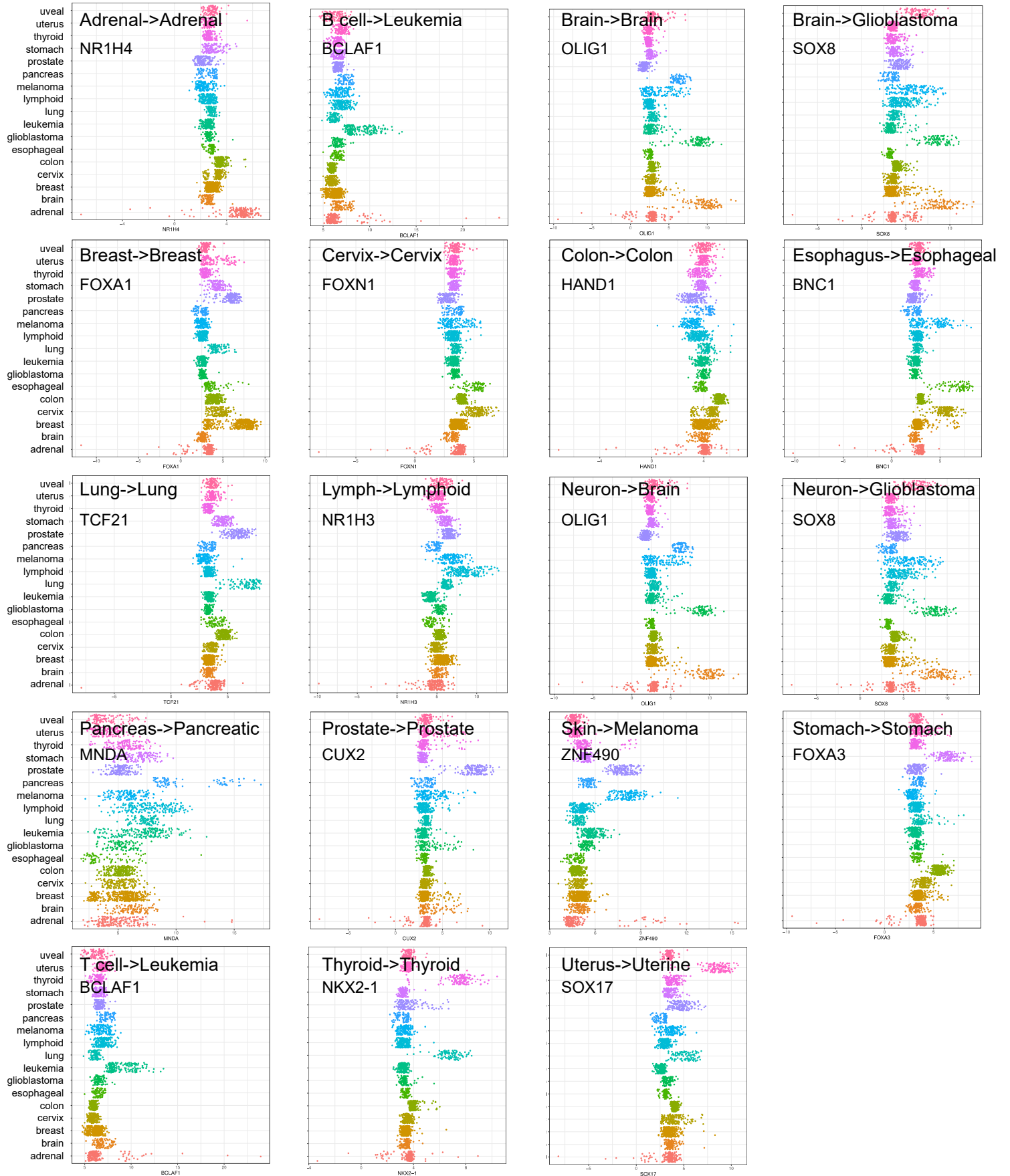

Figure S3

Supplement: Supplementary file 1 [file cancers-15-04167-s001.zip › FigureS3.pdf]

# ENRICHR: ChEA 2022 UMAP (query->target)

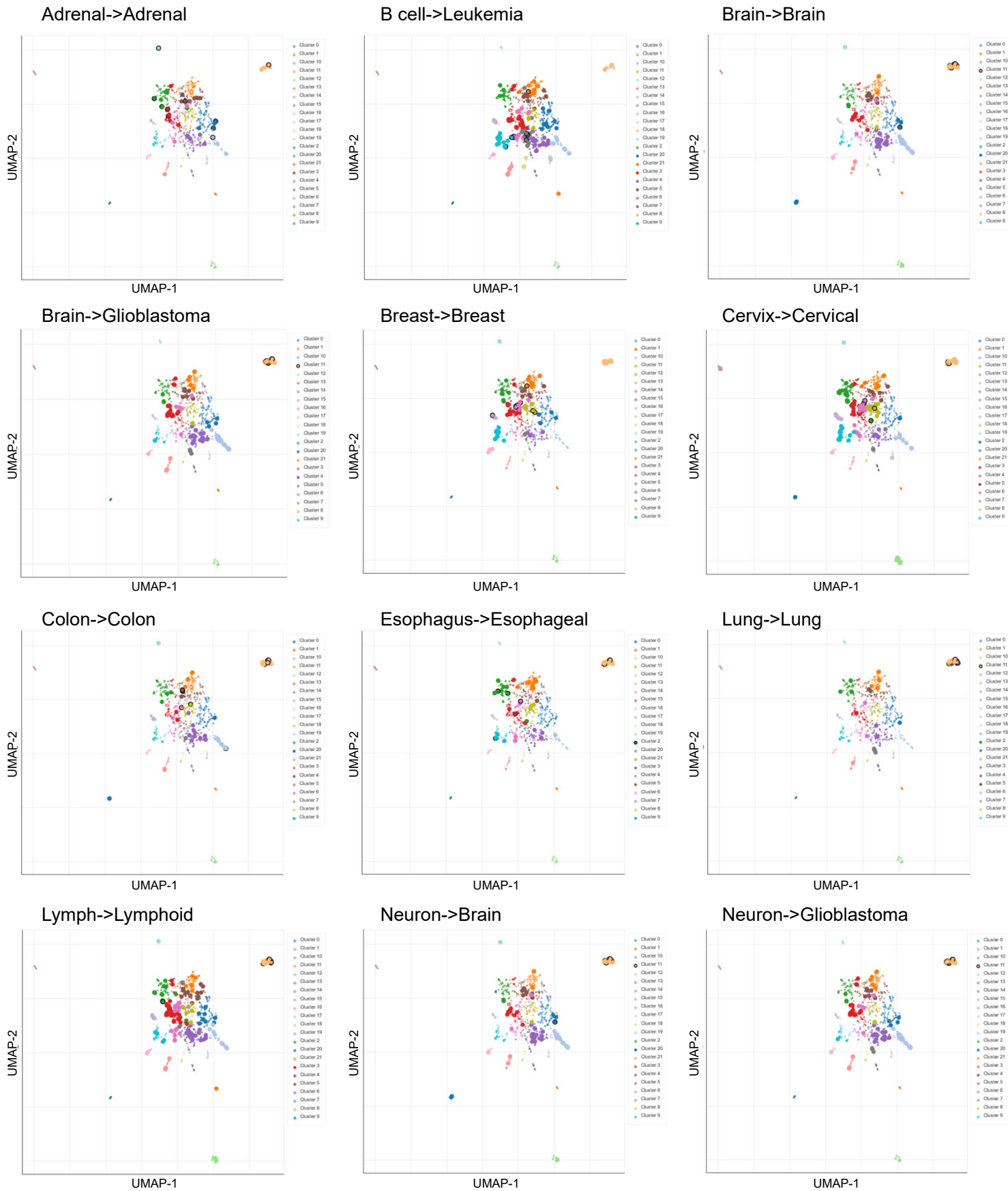

Figure S4

Supplement: Supplementary file 1 [file cancers-15-04167-s001.zip › FigureS4.pdf]

ENRICHHR: ChEA 2022 UMAP (query->target)

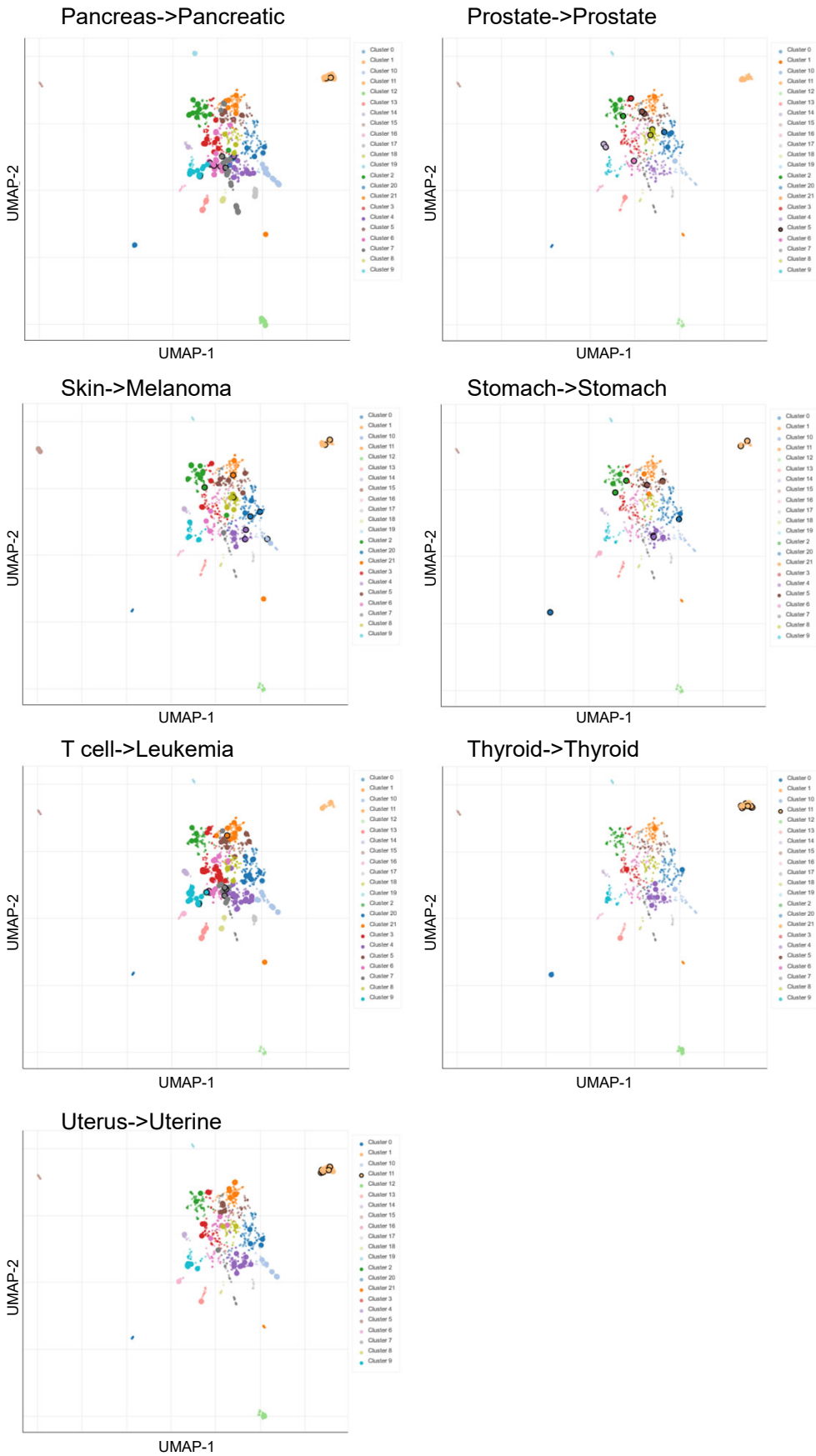

Figure S5

Supplement: Supplementary file 1 [file cancers-15-04167-s001.zip › FigureS5.pdf]

# ENRICHR: ChEA 2022 Bar Chart (query->target)

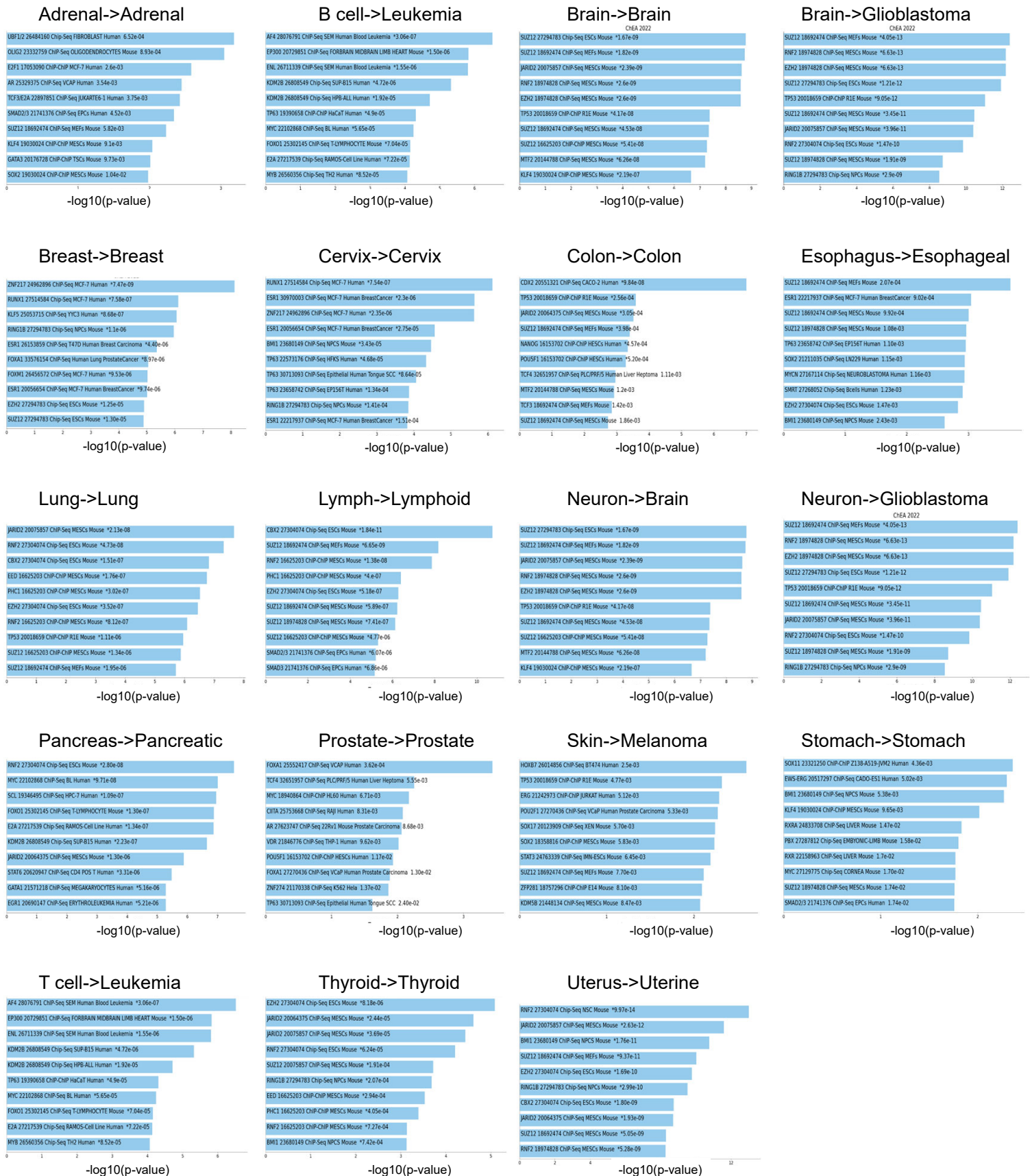

Figure S6

Supplement: Supplementary file 1 [file cancers-15-04167-s001.zip › FigureS6.pdf]

### ENRICH: ChEA 2022 clustergram (query->target)

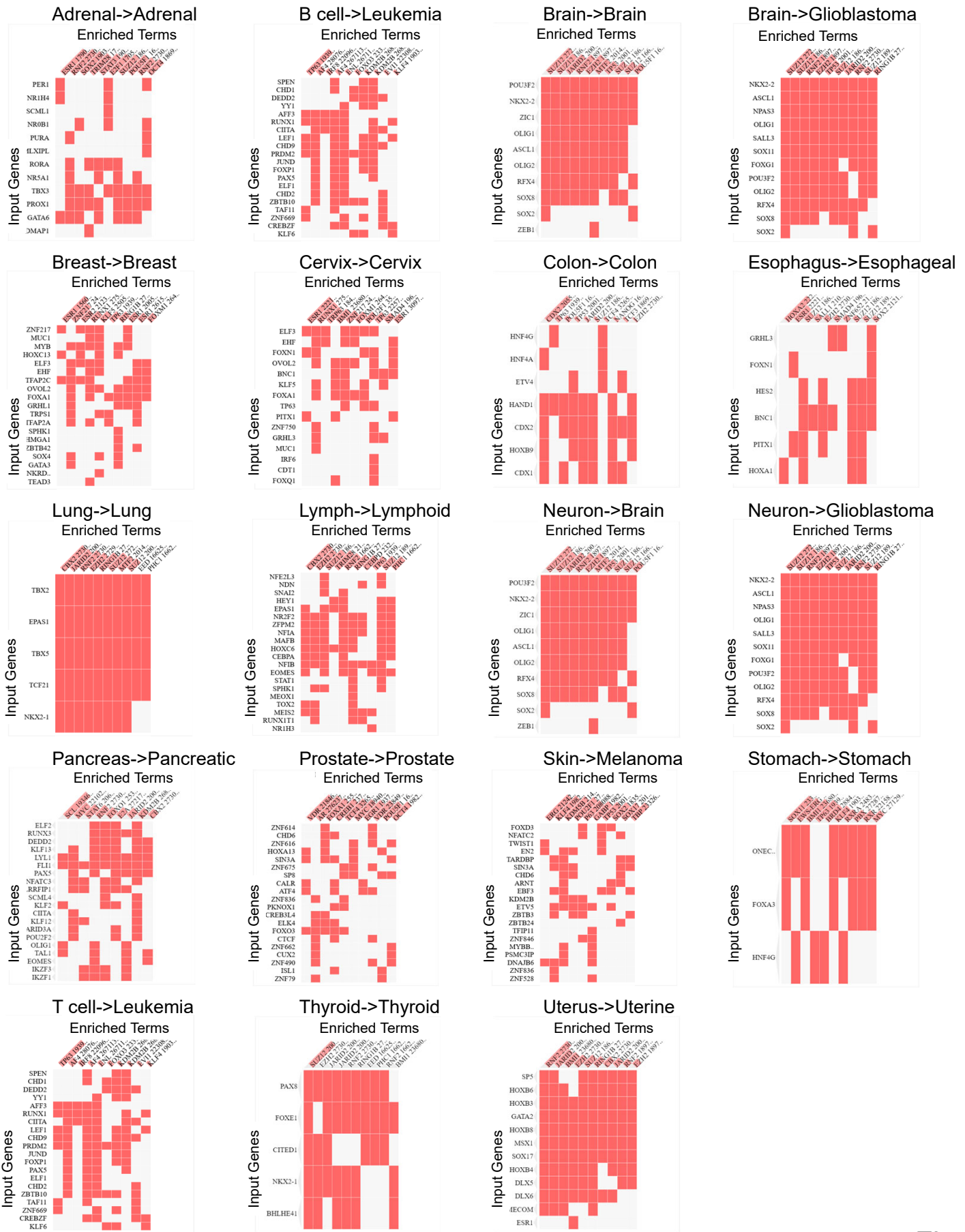

Figure S7

Supplement: Supplementary file 1 [file cancers-15-04167-s001.zip › FigureS7.pdf]

**A**

**Lung**

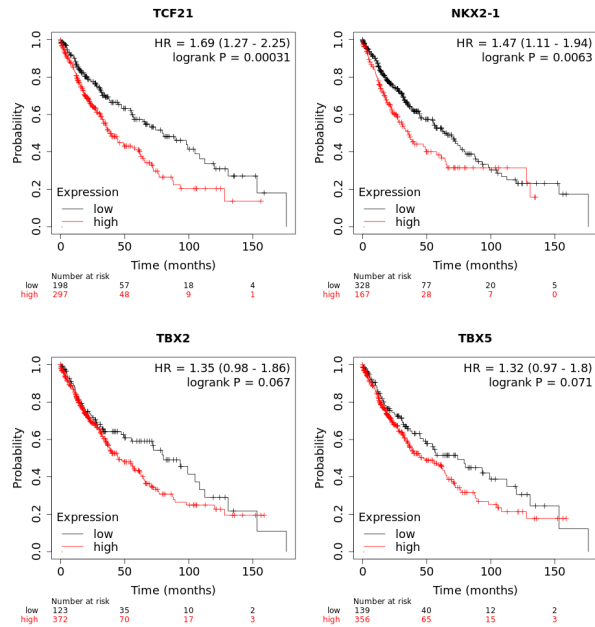

**B**

**Pancreatic**

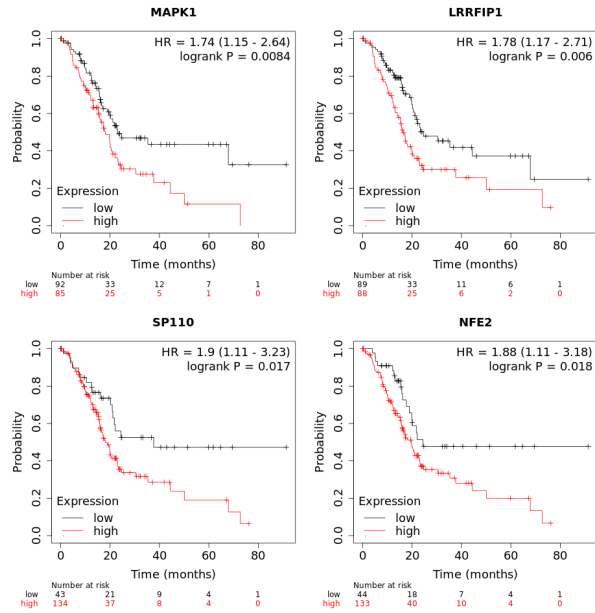

Figure S8

Supplement: Supplementary file 1 [file cancers-15-04167-s001.zip › FigureS8.pdf]
